# Prevalence of Self-Medication Among Female University Students During Examinations: A Cross-Sectional Study in Saudi Arabia

**DOI:** 10.7759/cureus.37269

**Published:** 2023-04-07

**Authors:** Lama Hamad M Alomaim, Ameera Faleh Alnefaie, Norah Abdullah Alowaymir, Nouf Abdulrahman Saleh Alahedb, Hailah Omar A Alomair, Rana Saud M Alanazi, Lamya Zaid Dakheel Alanazi, Haya Abdullah Naif Alshalawi, Tarfa Albrahim

**Affiliations:** 1 College of Health and Rehabilitation Sciences, Department of Health Sciences, Clinical Nutrition, Princess Nourah Bint Abdulrahman University, Riyadh, SAU

**Keywords:** self-medication, saudi arabia, self-care management, female college students, over-the-counter drugs

## Abstract

Introduction

Those who practice self-care using over-the-counter (OTC) products believe that these medications are relatively safe. They can be used to treat mild illnesses that do not require medical consultation. However, improper self-medication using OTC medicines because of inadequate knowledge of their side effects and interactions can result in drug-related issues and even death. The current study was performed using the foundation year students of Princess Nourah Bint Abdul Rahman University (PNU) as subjects, to examine the use of OTC medicines during examination times.

Methods

This cross-sectional study was done on 213 (response rates 87.7%) foundation-year female students in the Health Colleges at PNU in Riyadh. Using a 26-item, self-administered, online questionnaire, data were collected.

Results

It was found that more than 50% of the students used OTC medicines habitually during exam periods. A majority (90.6%; p< 0.0001) of the students mentioned that the overuse of Panadol Extra was very safe while 67.6% (p< 0.0001) of them declared that nonsteroidal anti-inflammatory drugs (NSAIDs) would not induce stomach ulcer formation. A higher percentage (67.6%; p<0.0001) of the students confirmed using OTC medicines during exam time for headache relief. A higher percentage (72.8%; p< 0.0001) of the students indicated that because the OTC medications were readily available and they believed these drugs were safe, they used sizable quantities during the time of the examinations. Furthermore, 69% (p< 0.0001) of the students confessed that because of a friend’s recommendations, they started trying OTC medicine. Above 67% (p< 0.0001) stated that OTC medications are inexpensive and easily available in Saudi Arabia.

Conclusion

To conclude, the findings of this study reiterated the high usage of OTC medicines by female students during the time of examination, and the highest used were painkillers.

## Introduction

Across the world, the practice of using over-the-counter (OTC) drugs for self-medicating is more prevalent than the use of prescription drugs [1-3]. According to the World Health Organization (WHO) specifications, both developed and developing countries reveal that the practice of self-medication has become an essential part of daily life and plays a vital part in the healthcare system as the socio-economic status and education levels of the population continue to improve [4,5]. In the event of the most common illness, self-medication appears to be the preferred path, making the practice of self-medication globally very popular [6]. In general, non-prescription drugs or OTC medications include those medications that can be procured without a doctor's prescription. These medicines have high efficacy and are safe to use when the guidelines on the label are followed or when a healthcare specialist directs their usage [7]. Per prior research work, globally, self-medication is highly prevalent and falls anywhere in the range of 11.2% to 93.7% based on the population targeted and the country in which it occurs [1,8].

For those practicing self-care, OTC drugs are considered quite safe, especially in the treatment of mild indispositions that do not necessitate medical consultation; however, any improper self-medication with OTC drugs arising from a paucity of knowledge regarding their side effects and interactions can result in drug-related issues with serious repercussions and even death [9-11]. A previous study reported that the most common reasons cited for self-medication include the greater convenience of going to a pharmacy than consulting with a doctor and a means of circumventing a hospital visit to receive treatment [12]. On the other hand, a sharp rise in the popularity of self-medication is a serious issue that healthcare decision-makers and policy-makers have to grapple with [6].

Studies done in different countries on self-medication practices targeted college students. They reported a high incidence of this practice in this specific population [13,14]. During exam time, the person may take nonprescription drugs to help him or her focus, rest, or alter their psyche [14,15]. For comparison, when studies on the prevalence of using OTC medications, particularly among female students are compared in developed countries, data are still inadequate because of the lack of relevant investigations being done in these specific categories. Hence, the present study aimed to find out the level of self-medication practice among female university students in Saudi Arabia.

## Materials and methods

Study design and setting

A descriptive cross-sectional study was performed on 213 female students (aged 18-21 years) belonging to the foundation year in the Health College at the Princess Nourah Bint Abdulrahman University (PNU) in Riyadh. The students' demographic data and knowledge of OTC medications were assessed.

Ethical considerations

This research was conducted in adherence with the Declaration of Helsinki guidelines and its approval, as well as that of the Institutional Review Board committee of PNU. All participants signed the online informed consent form by clicking “I give consent to participate” in this study. A positive response opened the study questionnaire for the participants. All participation was voluntary, and all participants were given the option to decline to join the study. Identification-related information (like name, address, etc.) was not required to maintain participant confidentiality. The study poses no more than minimal risk to the participants. Therefore, the study has been deemed exempt from IRB review of Princess Nourah Bint Abdulrahman University (PNU) Riyadh, Saudi Arabia (IRB Log Number: 23-0204).

Inclusion and exclusion criteria

The study included female students of the foundation year of PNU, aged 18-21 years, Students of other colleges were not included. Students who did not consent to participate and incomplete questionnaires were excluded from the study.

Study instruments

The objectives of the study were met by gathering data through the use of a 26-item questionnaire [16]. Primarily, four sections were included in the questionnaire.

Section One

This section was included mainly to record the demographic information of the participants, namely, age and year of study.

Section Two

In this section, the aim was to evaluate the degree of general knowledge the students have regarding the safety and toxicity of OTC medications. A nominal scale (Yes/No) was used to assess eight items. The knowledge levels of each respondent were clearly distinguishable. For every correct answer, one mark was added to the total score of each respondent; no marks were awarded for an incorrect answer; eight was the maximum possible score that could be attained. The respondents were awarded one mark for choosing the “No” option for the first statement (reverse scoring was done). “Yes” was the correct answer for the remaining seven statements. Students received one score for yes responses. To more succinctly clarify the difference in the knowledge level among the participants in this study, the knowledge score was further distinguished into three classes: 0-3 (Poor Level of Knowledge), 4-6 (Moderate Level of Knowledge), and 7-8 (Good Level of Knowledge).

Section Three

In this section, the views of the participants on the sale and consumption of OTC products in Saudi Arabia were assessed. Six items were given and on a two-item nominal scale, the respondents needed to express their concepts (Yes/No).

Section Four

The central section of the present study tool estimates the use of OTC drugs by the participants during the time of the examination. This section includes six statements that express the justification of the participant for their choice of using OTC substances during the exam period. The first five statements included two response options (Yes/No). The last question (number six) was open-ended and requested the kind of OTC medication the students used during the exams. The answers to the first five statements were scored and the use of OTC medications among the study population was then analyzed.

Statistical analysis

The data were analyzed using IBM SPSS Statistics (Version 23; IBM Corp, Armonk, NY). For categorical variables, the data were represented as numbers and percentages. The chi-square test was used to compare the evaluation of the degree of knowledge about the safety of using OTC products, knowledge regarding the use of such medications during exam time, and the opinions of the participants about the purchase and use of the OTC products during the time of the exams, The overall attitude of the students toward the use of over-the-counter medications during exams is shown as mean ± SD. The significance was selected as p<0.05.

## Results

Table 1 lists the knowledge levels of the study population regarding the safety of using OTC medications. A high percentage (90.6%) of students indicated the safety of using Panadol Extra during exam time (p<0.0001); 39% stated that the use of herbal/natural products for memory and attention would not have any ill effects on the human body (p<0.001); 84% cited serious side effects as the outcome of the long-term use of OTC medications. A little over half the study population (56.8%) acknowledged ignorance about the overuse of Panadol (paracetamol) and did not know it could lead to liver toxicity; around one-third of the participants (32.4%) responded that stomach ulcers (p<0.0001) could result from the use of nonsteroidal anti-inflammatory drugs (NSAIDs; ibuprofen) while a little above half of the study population (57.3%) revealed that the misuse of NSAIDs (ibuprofen) could damage the kidneys. It was encouraging that around 70.4% of the students read the medication leaflet prior to using any medication (p<0.0001); in fact, 95.8% mentioned that the best way to lower the risk of severe side effects from antihistamines (flu medications) would be for the users to exercise caution and take the doses according to the directions given (p<0.0001).

**Table 1:** Respondent's knowledge of the safety of over-the-counter (OTC) medicine Responses were compared using the chi-square (nonparametric) test. p-values of <0.05 are considered statistically significant.

Questions	Response	N (%)	p-value
Overusing Panadol Extra is very safe during the exam period	No	20 (9.4%)	< 0.0001
	Yes	193 (90.6%)	
The use of herbal/natural products for memory and attention will not cause negative effects on the human body	No	130 (61%)	< 0.001
	Yes	83 (39%)	
Long-term use of OTC medications will cause serious side effects	No	34 (16%)	< 0.0001
	Yes	179 (84%)	
Overusing Panadol (paracetamol) will cause liver toxicity	No	121 (56.8%)	0.047
	Yes	92 (43.2%)	
NSAIDs (ibuprofen) cause stomach ulcers	No	144 (67.6%)	< 0.0001
	Yes	69 (32.4%)	
NSAIDs (ibuprofen) may cause harm to your kidneys if they are misused	No	91 (42.7%)	< 0.034
	Yes	122 (57.3%)	
I read the medication leaflet before using any medication	No	63 (29.6%)	< 0.0001
	Yes	150 (70.4%)	
To decrease the risk of serious side effects from antihistamines (flu medications), users should carefully follow all dosage directions	No	9 (4.2%)	< 0.0001
	Yes	204 (95.8%)	

**Figure 1:**
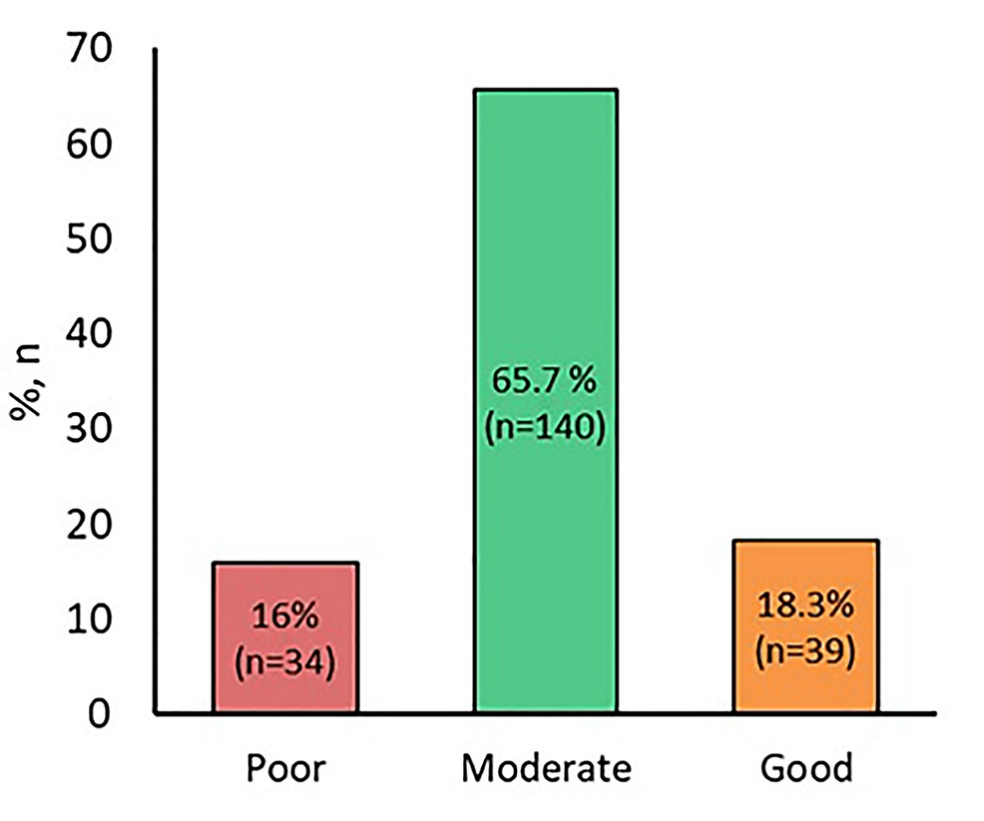
Overall knowledge score of over-the-counter medicines among students

The scores of the overall level of knowledge regarding OTC medicine in the student population can be seen. A majority of the respondents reveal a moderate level of knowledge (> 65% of students); next, 18.3% show a good level of knowledge, while just 16% of the student population reveal poor knowledge regarding OTC medicine (Figure 1).

As seen in Table 2, the degrees of knowledge regarding the use of OTC products during the exams are listed. Above half the student population (51.6%) utilized OTC medicines during the time of the exam (p<0.631) while 67.6% used OTC medicine during the examinations for headache relief (p<0.0001). A greater percentage of the students (63.8%) mentioned that several colleagues of theirs took OTC products too (p<0.0001). Only 50.7% took Panadol Night during the exam times for the relief of mild to moderate pains like headaches, which induced sleep disturbances; finally, 76.6% of the student population consumed energy drinks during the time of the examination (p< 0.0001).

**Table 2:** Respondents' use of over-the-counter (OTC) products during the exam Responses were compared using the chi-square (nonparametric) test. p-values of <0.05 are considered statistically significant.

Questions	Response	N (%)	P-value
I usually use OTC medicines during the exam period	No	103 (48.46%)	0.631
	Yes	110 (51.6%)	
I use OTC medicines during the exam period to relieve my headache	No	69 (32.4%)	< 0.0001
	Yes	144 (67.6%)	
I note the use of OTCs by my colleagues	No	77 (36.2%)	< 0.0001
	Yes	136 (63.8%)	
I use Panadol Night to relieve mild to moderate pain, such as headaches, that cause sleeping disturbance during my exam period	No	105 (49.3%)	0.837
	Yes	108 (50.7%)	
I use energy drinks during the exam period	No	161 (76.6%)	< 0.0001
	Yes	52 (24.4%)	

Table 3 lists the opinions of the students in the study, with respect to the purchase and use of OTC products during exam time. Quite a high percentage of students (67.1%) cited the affordability and ready availability of OTC medications in Saudi Arabia, which are the main reasons for the popular usage of these OTC medicines (p<0.0001). Below half of the student population (40.4%) acknowledged that advertising was the primary reason for the usage of OTC medicines. However, 53.5% (p = 0.304) of the students were comfortable with using OTC products because they consulted with the pharmacists prior to purchase and use. A larger number of the students (59.6%) lacked knowledge regarding the sedative result of antihistamines (flu medications), which resulted in misuse, especially during the time of the examinations. Besides, 69% of students stated that during exam time, they used medicines in response to suggestions made by a friend (p<0.0001) while 72.8% of students stated that as the OTC medications were readily available, and they believed these were safe to use, many of them consumed plenty of these drugs during the time of examinations.

**Table 3:** Respondents' views toward the use and purchase of over-the-counter (OTC) products during exams Responses were compared using the chi-square (nonparametric) test. p-values of <0.05 are considered statistically significant.

Questions	Response	N (%)	P-value
OTC medications are cheap and easily available in Saudi Arabia, which is why all people use them	No	70 (32.9%)	< 0.0001
	Yes	143 (67.1%)	
Advertising is the main cause that people use OTC medicines	No	127 (59.6%)	0.005
	Yes	86 (40.4%)	
I feel convenient with the use of OTC medicines because of the consultation provided by the pharmacist	No	99 (46.5%)	0.304
	Yes	114 (53.5%)	
The sedative effect of antihistamines (flu medications) makes people misuse them typically during the exam period	No	127 (59.6%)	0.005
	Yes	86 (40.4%)	
During the exam period, many try medicines according to a friend's suggestion	No	66 (31%)	< 0.0001
	Yes	147 (69%)	
The availability of OTC medicines and the belief in their safety leads me to use them a lot during the exam period	No	58 (27.2%)	< 0.0001
	Yes	155 (72.8%)	

Table 4 lists the students' attitudes to the usage of over-the-counter products during examinations, age-wise (mean ± SD). However, the differences observed were not significant between students younger than 19 years and those older than 19 years in terms of their knowledge regarding the safety of OTC medicines (p = 0.644), their consumption of OTC medications during the time of exams (p = 0.167), and their opinions regarding the purchase and usage of OTC medicines during the exam times (p = 0.621).

**Table 4:** Age-wise students' attitude toward the use of over-the-the-counter (OTC) medicines during exams (mean ± SD) Responses were compared using the chi-square (nonparametric) test. p-values of <0.05 are considered statistically significant.

Students' attitudes toward the use of over-the-counter medicines during exams	Age groups	Mean ± SD	P-value
Knowledge about the safety of OTC medicine	<19 Years	5.07 ± 1.41	0.644
	≥ 19 Years	5.18 ± 1.50	
Respondent's use of OTC products during the exam	<19 Years	3.37 ± 1.50	0.167
	≥ 19 Years	3.48 ± 1.53	
Respondent's view toward the use and purchase of OTC products during exams	<19 Years	2.44 ± 1.38	0.621
	≥ 19 Years	2.65 ± 1.47	

**Figure 2:**
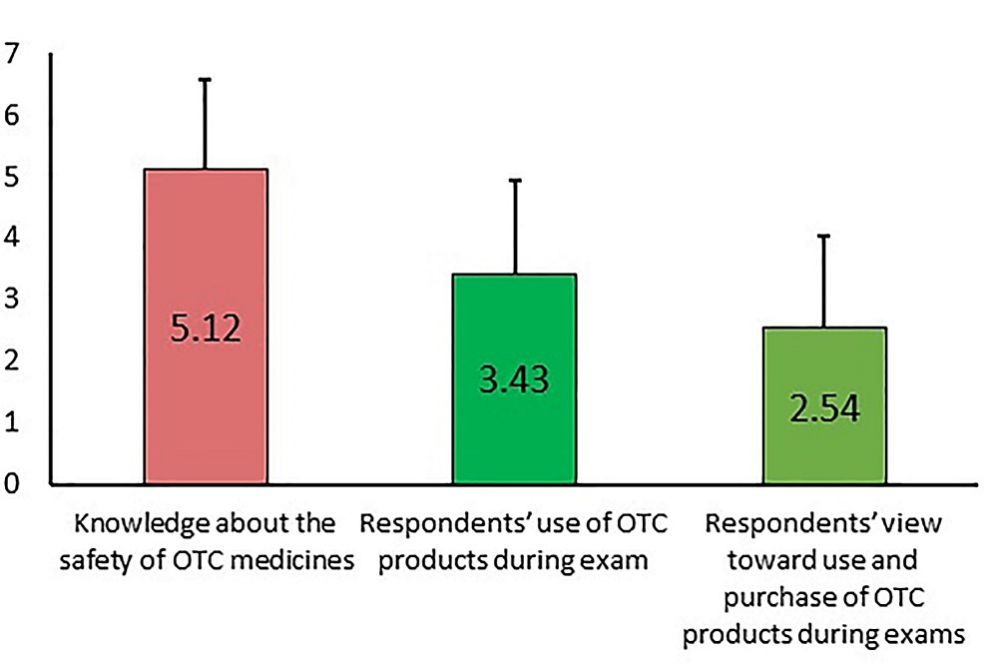
Students’ attitude toward the use of over-the-counter (OTC) medicines during exams (mean ± SD)

Figure 2 shows the mean levels of attitudes of the student population regarding the use of over-the-counter medications during the examination period. A higher mean level (5.12) was noted in terms of knowledge regarding the safety of the use of OTC products; a level of 3.43 was observed in the usage of OTC medicines by the participants during the time of the exam; a level of 2.45 was seen in the view of the respondents about the purchase and usage of OTC substances during the exam periods.

## Discussion

The current cross-sectional study was conducted to assess the incidence and determinants of self-medication using 213 female university students of PNU in Riyadh, Saudi Arabia. The principal findings of the present study revealed that 51.6% of the students normally used OTC medications during exam time. Our results demonstrated a lowered percentage of comprehension when compared with the findings of earlier studies performed in other Saudi Arabian universities [17,18]. These studies indicated that overall, the prevalence of self-medication among female students was 98.2% in Hail, Saudi Arabia, and the most often cited medical reasons that induced such self-medication practices were headaches (92.85%); after that came cough (37.5%), colic (31.9%), and influenza (30.3%) [13]. In Najran University, Saudi Arabia, reports from another recent study showed 60% as the overall prevalence of self-medication in the study population [18]. Besides, the most common medical conditions leading to self-medication were identified as headache (65.9%), fever (30.2%), and cold/flu (31.2%) [18]. Most of the participants (84%) in this study cited that serious side effects would arise from the long-term use of OTC medications and many (90.6%) disagreed regarding the safety of Panadol Extra overuse during the time of examination; one reasonable explanation for the low prevalence rate between the present study and other studies may be the higher knowledge level regarding the safety of consuming OTC medicines. Furthermore, in one study from Saudi Arabia, it appeared that female students were found to be more knowledgeable regarding the safety issues linked to the use of OTC products than male students [16]. One more possible reason for the decrease might be the multiple factors that affected the outcomes of the study, namely, sample size, age, and gender (only female).

The findings of the current study indicated that most students take OTC medicines during the time of exam for headache relief. This outcome concurred with the findings of an earlier study conducted among university students in Saudi Arabia, in which the most popular reason for following self-medication practices during examination time was for the treatment of headaches [17,18]. Usually, university students face constant pressure as they work hard to accomplish their goals, which puts them at considerable risk of contracting minor ailments, which, coupled with their busy social life, could raise the necessity for the intake of OTC medications [19].

Regarding the rise in the usage of OTC medications, the present study also estimated the opinions of the students regarding the purchase and use of OTC medications during the time of examination. The participants were asked multiple questions regarding the different reasons for the usage of OTC products. Most of the students provided three reasons. First, 72.8% of the students mentioned that because the OTC medicines were easily available and because of their belief that these drugs were safe, they were used a lot during the time of the examinations. Second, 69% of the students cited that it was a recommendation or suggestion by friends that induced them to take such medicines during exam time. Third, 67% of the students stated that because the OTC medications in Saudi Arabia were readily available and inexpensive, many opted to utilize them. These results concur with the findings of earlier studies, which report the main reasons that could affect OTC medication usage are as follows: they are easy to access, advertising is aggressive, and pharmacist consultations are readily available [16]. Further studies revealed that the lack of regulation or restrictions in gaining access to OTC medications is one of the chief reasons for the misuse of these substances [20]. Moreover, in developing countries, the improper use of these medications is high because of the inadequate knowledge of the students regarding them [21]. In the present study, it is evident that the major part of the participant population possessed only moderate knowledge (> 65% of students had reasonable knowledge), followed by 18.3% who possessed a good level of knowledge, and only 16% students had poor knowledge of OTC. Furthermore, above 90% of students expressed that it was very safe to use Panadol Extra during the examination period while 67.6% of the students mentioned that NSAIDs (ibuprofen) would not induce the formation of stomach ulcers, clearly revealing the lack of knowledge of the students regarding some of the effects of self-medication. In the earlier findings, poor knowledge levels about self-medication were mentioned, which corresponded to the findings of other international studies done with university students [22-24]. Despite the misuse of OTC medications and the resulting medicine-related issues, their number in the market and the prevalence of usage are escalating in certain countries [2].

A few limitations are observed in this study, namely, the small sample size, analysis of limited demographic data, the use of only a single center to conduct the study, and restriction of the study to only female students. These can be circumvented by performing the same study on a larger scale.

## Conclusions

In conclusion, the findings of this study clearly showed the widespread usage of over-the-counter medications among the student population during examination time, with painkillers being the most used. Therefore, the urgent need to provide sufficient evidence for the burden is important. It is recommended that campaigns or educational programs be developed to provide adequate information regarding the ill effects of utilizing non-prescribed, over-the-counter medications. The present study offers valuable data regarding self-medication and its prevalence among the female university student population, particularly at the time of examinations in Saudi Arabia.

## References

[1] Chautrakarn S, Khumros W, Phutrakool P (2021). Self-medication with over-the-counter medicines among the working age population in metropolitan areas of Thailand. Front Pharmacol.

[2] Mirdad OA, Esheba GE, Mousa AH (2023). Over-the-counter medication use among parents in Saudi Arabia. Int J Environ Res Public Health.

[3] Al-Ghamdi S, Alfauri TM, Alharbi MA (2020). Current self-medication practices in the Kingdom of Saudi Arabia: an observational study. Pan Afr Med J.

[4] Shah K, Halder S, Haider SS (2021). Assessment of knowledge, perception, and awareness about self-medication practices among university students in Nepal. Heliyon.

[5] Thomas D, Zachariah S (2018). Social and Administrative Aspects of Pharmacy in Low-and Middle-Income Countries. Present Challenges and Future Solutions. https://www.sciencedirect.com/book/9780128112281/social-and-administrative-aspects-of-pharmacy-in-low-and-middle-income-countries?via=ihub=.

[6] Shalini A, Logaraj M (2021). Prevalence and determinants of self medication use among the adult population residing in a sub urban areas near Chennai, Tamil Nadu. J Family Med Prim Care.

[7] (2022). U.S. Food and Drug Administration. Understanding over-the-counter medicines. https://www.fda.gov/drugs/buying-using-medicine-safely/understanding-over-counter-medicines.

[8] Abdi A, Faraji A, Dehghan F, Khatony A (2018). Prevalence of self-medication practice among health sciences students in Kermanshah, Iran. BMC Pharmacol Toxicol.

[9] de Oliveira AV, Rocha FT, Abreu SR (2014). Acute liver failure and self-medication. Arq Bras Cir Dig.

[10] Austin AE, Proescholdbell SK, Creppage KE, Asbun A (2017). Characteristics of self-inflicted drug overdose deaths in North Carolina. Drug Alcohol Depend.

[11] Ocan M, Obuku EA, Bwanga F, Akena D, Richard S, Ogwal-Okeng J, Obua C (2015). Household antimicrobial self-medication: a systematic review and meta-analysis of the burden, risk factors and outcomes in developing countries. BMC Public Health.

[12] Oleszkiewicz P, Krysinski J, Religioni U, Merks P (2021). Access to medicines via non-pharmacy outlets in European countries—a review of regulations and the influence on the self-medication phenomenon. Healthcare (Basel).

[13] Alshammari F, Alobaida A, Alshammari A (2021). University students’ self-medication practices and pharmacists’ role: a cross-sectional survey in Hail, Saudi Arabia. Front Public Health.

[14] Bekele KM, Abay AM, Mengistu KA, Atsbeha BW, Demeke CA, Belay WS, Yimenu DK (2020). Knowledge, attitude, and practice on over-the-counter drugs among pharmacy and medical students: a facility-based cross-sectional study. Integr Pharm Res Pract.

[15] Conca AJ, Worthen DR (2012). Nonprescription drug abuse. J Pharm Pract.

[16] Almalak H, Albluwi AI, Alkhelb DA, Alsaleh HM, Khan TM, Hassali MA, Aljadhey H (2014). Students' attitude toward use of over the counter medicines during exams in Saudi Arabia. Saudi Pharm J.

[17] Orayj K, Alshahrani SM, Alqahtani AM, Alasmari N, Atef AA, Jrais HS, Muslot D (2021). The use of over-the-counter (OTC) medications by university students during examinations in Saudi Arabia: a cross-sectional study. Risk Manag Healthc Policy.

[18] Al-Qahtani AM, Shaikh IA, Shaikh MA, Mannasaheb BA, Al-Qahtani FS (2022). Prevalence, perception, and practice, and attitudes towards self-medication among undergraduate medical students of Najran University, Saudi Arabia: a cross-sectional study. Risk Manag Healthc Policy.

[19] Birru EM, Abay Z, Abdelwuhab M, Basazn A, Sirak B, Teni FS (2016). Management of headache and associated factors among undergraduate medicine and health science students of University of Gondar, North West Ethiopia. J Headache Pain.

[20] Manohar HD, Manohar HL (2015). Impact of knowledge and attitude on practices of over the counter medications. IEOM Soc.

[21] Tesfamariam S, Anand IS, Kaleab G, Berhane S, Woldai B, Habte E, Russom M (2019). Self-medication with over the counter drugs, prevalence of risky practice and its associated factors in pharmacy outlets of Asmara, Eritrea. BMC Public Health.

[22] Gyawali S, Shankar PR, Poudel PP, Saha A (2015). Knowledge, attitude and practice of self-medication among basic science undergraduate medical students in a medical school in western Nepal. J Clin Diagn Res.

[23] Mitra AK, Imtiaz A, Al Ibrahim YA, Bulbanat MB, Mutairi MFA, Musaileem SFA (2019). Factors influencing knowledge and practice of self-medication among college students of health and non-health professions. IMC J Med Sci.

[24] Sambakunsi CS, Småbrekke L, Varga CA, Solomon V, Mponda JS (2019). Knowledge, attitudes and practices related to self-medication with antimicrobials in Lilongwe, Malawi. Malawi Med J.

